# Supplementary figures and images for: Impact of sample processing delays on plasma markers of inflammation, chemotaxis, cell death, and blood coagulation
Source: PLoS One. 2024 Oct 31;19(10):e0311921. doi: 10.1371/journal.pone.0311921 (PMC11527306; doi:10.1371/journal.pone.0311921)

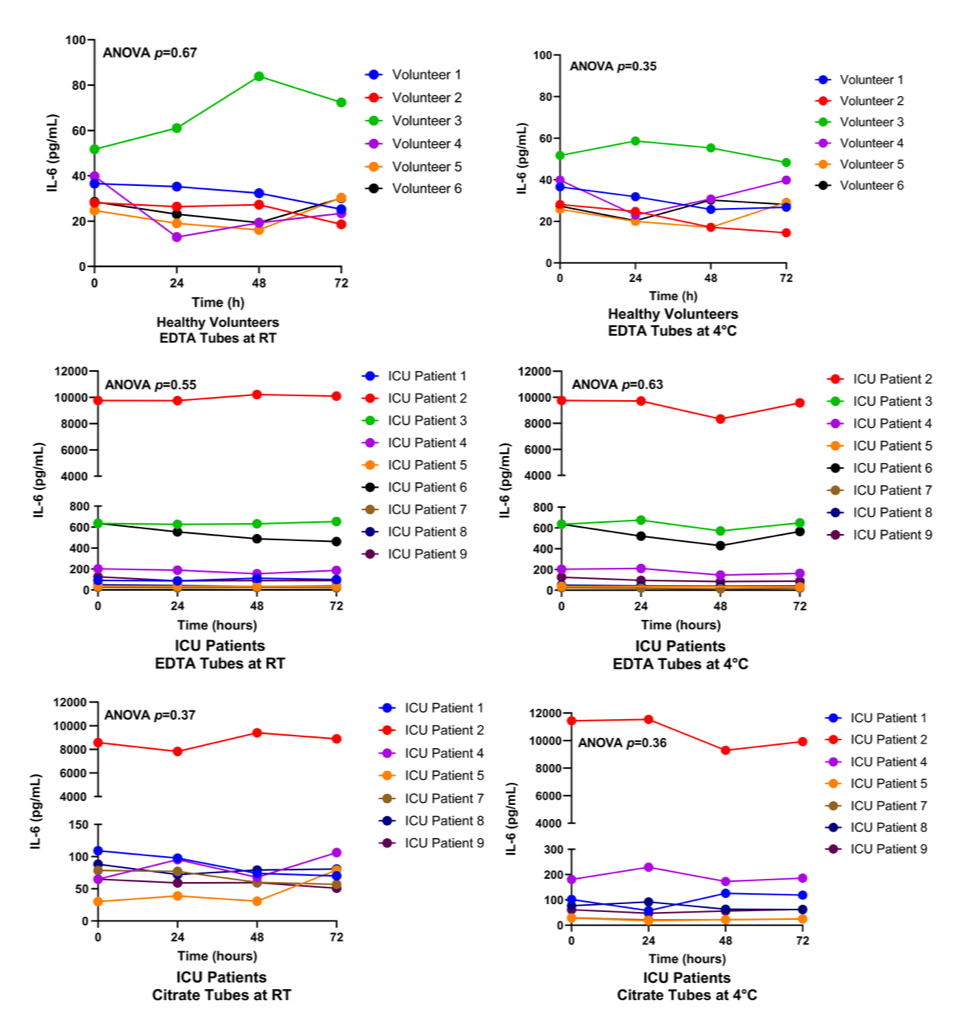

Supplement: S1 Fig — Blood was collected from healthy volunteers (n = 7) and ICU patients (n = 9) into citrate or EDTA tubes. The blood was stored at RT or 4°C for 0, 24, 48, or 72 hours. No significant changed were observed in IL-6 levels with delayed processing conditions at RT or 4°C. (TIFF) [file pone.0311921.s001.tiff]

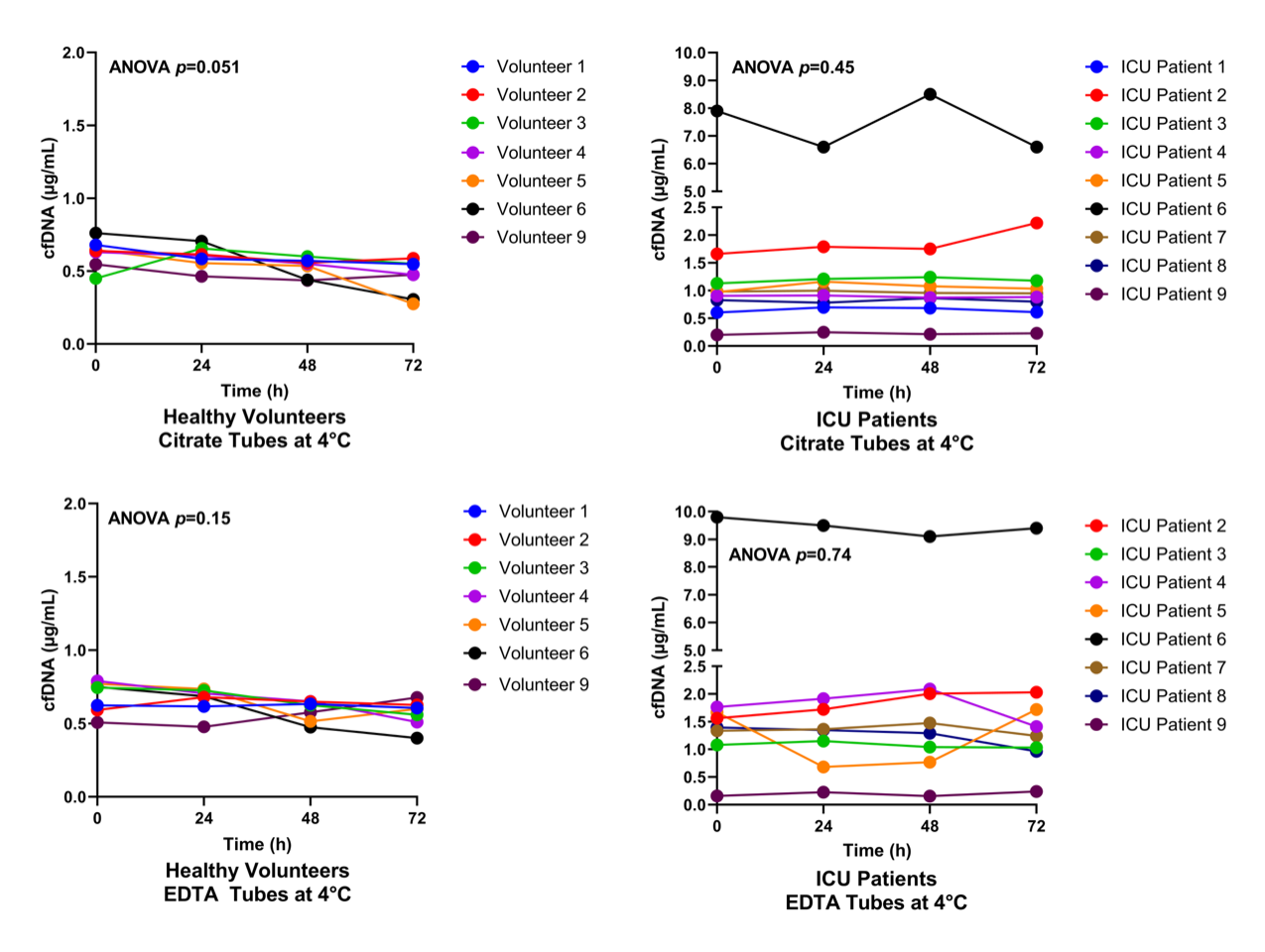

Supplement: S2 Fig — (TIFF) [file pone.0311921.s002.tiff]
