# Supplementary material for: Impact of sample processing delays on plasma markers of inflammation, chemotaxis, cell death, and blood coagulation
Source: PLoS One. 2024 Oct 31;19(10):e0311921. doi: 10.1371/journal.pone.0311921 (PMC11527306; doi:10.1371/journal.pone.0311921)
Supplement: S1 Table — AAA (abdominal aortic aneurysm); AKI (acute kidney injury); GI (gastrointestinal); ICH (intracranial hemorrhage); SOFA score (sequential organ failure assessment score); N.D. (not done). (PDF) [file pone.0311921.s003.pdf]

Supplemental Table 1

	P1	P2	P3	P4	P5	P6	P7	P8	P9
Sex	F	F	M	F	F	F	M	M	F
Admission Diagnosis	Trauma	Ruptured AAA	Sepsis	Trauma	AKI	GI Bleed	Trauma	ICH	Sepsis
SOFA score	1	8	3	7	4	5	1	0	3
Mechanical Ventilation	No	Yes	Yes	No	No	Yes	Yes	Yes	No
Vasopressors	No	Yes	Yes	No	Yes	Yes	Yes	Yes	Yes
Dialysis	No	No	No	No	Yes	No	No	No	No
Fibrinogen (g/L)	2.5	1.3	3.8	1.8	N.D.	N.D.	2.5	N.D	3.7
Lactate (mM)	5.9	5.7	2.0	2.2	0.6	13.4	2.6	2.5	2.0
WBC (10^9/L)	22.3	15.7	13.8	14.2	8.4	18.3	10.9	14.3	37.3
Platelet Count (10^9/L)	137	78	216	122	241	73	190	294	37
Antibiotics	No	Yes	Yes	Yes	No	Yes	Yes	No	Yes
Blood culture	N.D.	N.D.	*Klebsiella* pneumoniae	*Klebsiella aerogenes*	No growth	No growth	No growth	No growth	*Escherichia coli*
