# Supplementary material for: Impact of sample processing delays on plasma markers of inflammation, chemotaxis, cell death, and blood coagulation
Source: PLoS One. 2024 Oct 31;19(10):e0311921. doi: 10.1371/journal.pone.0311921 (PMC11527306; doi:10.1371/journal.pone.0311921)
Supplement: S2 Table — (PDF) [file pone.0311921.s004.pdf]

Supplemental Table 2
Multiplex cytokine levels in EDTA plasma samples from ICU patients

Sample ID	IFN-γ (pg/mL)	IL-6 (pg/mL)	IL-10 (pg/mL)	TNF (pg/mL)
Normal Range	0.46-22.8	0.12-0.99	0.09-2.71	0.31-2.32
P1-E-RT 0	1.82	45.06	5.85	0.18
P1-E-RT 24	1.24	41.64	6.17	0.24
P1-E-RT 48	below curve fit	42.81	5.13	0.43
P1-E-RT 72	0.96	42.02	5.08	1.25
P2-E-RT 0	21.08	9340.06	91.51	2.28
P2-E-RT 24	40.52	9790.77	90.93	1.89
P2-E-RT 48	18.67	9205.19	77.15	1.40
P2-E-RT 72	24.80	8647.18	65.46	1.69
P3-E-RT 0	4.25	319.82	174.40	15.46
P3-E-RT 24	5.19	310.56	166.59	13.09
P3-E-RT 48	2.59	330.59	162.67	13.07
P3-E-RT 72	2.85	328.43	162.21	12.56
P4-E-RT 0	1.43	73.70	99.95	1.93
P4-E-RT 24	3.68	67.16	90.73	1.70
P4-E-RT 48	1.76	59.79	86.00	1.73
P4-E-RT 72	0.84	61.00	85.60	1.82
P5-E-RT 0	7.99	6.38	0.42	0.58
P5-E-RT 24	7.87	6.13	0.37	0.43
P5-E-RT 48	9.35	6.82	0.41	0.78
P5-E-RT 72	8.37	6.77	0.33	0.74
P6-E-RT 0	23.72	282.15	12.02	2.62
P6-E-RT 24	18.25	265.71	12.06	2.58
P6-E-RT 48	15.65	249.65	10.66	2.02
P6-E-RT 72	15.28	232.54	9.32	2.09
P7-E-RT 0	below curve fit	18.20	0.65	0.06
P7-E-RT 24	1.43	16.26	0.79	0.08
P7-E-RT 48	1.17	16.11	0.71	0.16
P7-E-RT 72	1.89	17.36	0.88	0.27
P8-E-RT 0	3.99	22.48	1.75	0.39
P8-E-RT 24	3.36	15.56	1.68	0.32
P8-E-RT 48	2.66	14.63	1.47	0.57
P8-E-RT 72	4.31	13.60	1.34	0.72
P9-E-RT 0	6.69	36.88	5.32	3.78
P9-E-RT 24	6.50	37.69	5.53	4.31
P9-E-RT 48	5.44	35.24	5.05	4.19
P9-E-RT 72	4.44	34.95	4.50	4.47
