# Supplementary material for: Impact of sample processing delays on plasma markers of inflammation, chemotaxis, cell death, and blood coagulation
Source: PLoS One. 2024 Oct 31;19(10):e0311921. doi: 10.1371/journal.pone.0311921 (PMC11527306; doi:10.1371/journal.pone.0311921)
Supplement: S3 Table — (PDF) [file pone.0311921.s005.pdf]

Supplemental Table 3
Multiplex chemokine levels in EDTA plasma samples from ICU patients

Sample ID	MIP-1β (pg/mL)	IP-10 (pg/mL)	MIP-1α (pg/mL)	IL-8 (pg/mL)	MCP-1 (pg/mL)	MCP-4 (pg/mL)
Normal Range	7.99-153	102 - 676	11.3 - 651	347 - 2478	42.3 - 185	29.5 - 136
P1-E-RT 0	41.94	201.05	8.98	1634.16	188.77	41.70
P1-E-RT 24	45.78	227.65	8.06	1906.20	178.6	56.40
P1-E-RT 48	44.87	206.60	7.42	5524.06	161.71	54.84
P1-E-RT 72	54.99	252.40	8.34	24371.96	189.51	68.59
P2-E-RT 0	132.9	1659.81	31.93	1684.76	624.49	20.24
P2-E-RT 24	76.43	1657.09	31.75	1338.78	473.63	15.12
P2-E-RT 48	77.06	1595.51	31.47	1123.41	504.21	15.76
P2-E-RT 72	66.89	1958.50	27.66	1417.32	565.07	16.85
P3-E-RT 0	4283.75	1132.95	249.7	2080.44	2229.8	96.65
P3-E-RT 24	4234.76	1023.83	265.1	867.07	2425.13	127.49
P3-E-RT 48	4785.55	1296.50	257.52	677.86	3102.85	138.79
P3-E-RT 72	5333.61	1383.52	255.33	3881.68	3318.61	141.67
P4-E-RT 0	344.81	244.68	64.44	1213.31	758.29	42.13
P4-E-RT 24	252.18	237.87	59.19	1051.92	682.04	40.90
P4-E-RT 48	284.77	282.50	60.45	3087.35	721.50	42.79
P4-E-RT 72	287.90	323.67	74.51	314.39	850.03	48.62
P5-E-RT 0	76.16	210.66	20.49	1100.00	153.39	75.46
P5-E-RT 24	80.63	177.81	19.52	1546.84	118.13	74.86
P5-E-RT 48	99.23	190.60	20.54	677.86	121.02	82.00
P5-E-RT 72	108.52	194.43	25.19	1651.17	114.26	89.22
P6-E-RT 0	522.23	1700.22	42.71	1473.97	406.07	49.13
P6-E-RT 24	503.55	1387.69	41.80	1100.00	332.26	38.12
P6-E-RT 48	450.70	1400.75	44.79	1750.36	345.58	37.15
P6-E-RT 72	371.55	1366.94	43.80	1750.36	345.56	38.16
P7-E-RT 0	137.96	208.68	12.57	1436.41	53.41	16.87
P7-E-RT 24	132.41	200.20	10.39	1169.07	37.27	31.51
P7-E-RT 48	141.69	244.78	12.39	1950.95	38.69	47.13
P7-E-RT 72	140.99	202.09	13.3	1734.15	31.17	33.93
P8-E-RT 0	55.38	116.77	11.59	1528.89	42.72	19.58
P8-E-RT 24	78.57	125.70	11.28	1100.00	32.75	35.63
P8-E-RT 48	106.99	172.77	12.16	2163.22	37.96	53.70
P8-E-RT 72	123.74	169.43	13.93	1455.29	29.73	53.08
P9-E-RT 0	103.75	927.49	73.67	1766.45	165.28	30.92
P9-E-RT 24	105.09	816.81	67.05	1492.46	146.97	28.72
P9-E-RT 48	154.54	978.30	64.79	2052.24	143.37	31.73
P9-E-RT 72	183.94	894.97	68.45	1256.27	138.97	28.34
