# Supplementary material for: Impact of sample processing delays on plasma markers of inflammation, chemotaxis, cell death, and blood coagulation
Source: PLoS One. 2024 Oct 31;19(10):e0311921. doi: 10.1371/journal.pone.0311921 (PMC11527306; doi:10.1371/journal.pone.0311921)
Supplement: S4 Table — (PDF) [file pone.0311921.s006.pdf]

Supplemental Table 4
Cell-free DNA levels in plasma samples from ICU patients and healthy volunteers

cfDNA in ICU patients (µg/mL)										
	Time (h)	Patient 1	Patient 2	Patient 3	Patient 4	Patient 5	Patient 6	Patient 7	Patient 8	Patient 9
Citrate RT	0	0.60	1.66	1.13	0.91	0.97	7.7	0.98	0.83	0.20
	24	0.88	1.74	1.35	0.87	1.18	7.3	1.00	0.81	0.20
	48	0.62	1.82	1.17	0.90	1.04	8	1.00	0.94	0.22
	72	0.64	1.77	1.31	0.98	1.05	7.7	1.08	0.72	0.26
EDTA RT	0	0.94	1.56	1.08	1.76	1.67	9.8	1.33	1.40	0.16
	24	0.53	1.82	1.16	1.92	1.81	9.7	1.43	1.52	0.24
	48	0.51	2.03	1.41	2.07	1.93	9.4	1.61	1.53	0.24
	72	0.73	2.30	1.22	2.29	1.77	8.5	1.48	1.41	0.28
Citrate 4ºC	0	0.60	1.66	1.13	0.91	0.97	7.9	0.98	0.83	0.20
	24	0.70	1.79	1.21	0.91	1.16	6.6	1.00	0.78	0.25
	48	0.68	1.75	1.24	0.87	1.08	8.5	0.96	0.86	0.21
	72	0.61	2.22	1.18	0.88	1.03	6.6	0.95	0.80	0.23
EDTA 4ºC	0		1.56	1.08	1.76	1.67	9.8	1.33	1.40	0.16
	24		1.72	1.15	1.91	0.68	9.5	1.36	1.35	0.23
	48		2.01	1.04	2.09	0.77	9.1	1.48	1.29	0.16
	72		2.03	1.03	1.41	1.72	9.4	1.24	0.96	0.24

cfDNA in healthy volunteers (µg/mL)								
	Time (h)	Volunteer 1	Volunteer 2	Volunteer 3	Volunteer 4	Volunteer 5	Volunteer 6	Volunteer 9
Citrate RT	0	0.68	0.64	0.45	0.63	0.65	0.76	0.55
	24	0.61	0.61	0.65	0.71	0.64	0.64	0.51
	48	0.62	0.73	0.69	0.67	0.64	0.68	0.50
	72	0.59	0.58	0.61	0.61	0.44	0.39	1.26
EDTA RT	0	0.62	0.59	0.75	0.79	0.77	0.75	0.51
	24	0.73	0.68	0.87	0.76	0.66	0.66	0.63
	48	1.44	0.96	0.77	0.73	0.79	0.71	0.56
	72	0.89	1.12	0.80	0.60	0.53	0.65	0.66
Citrate 4ºC	0	0.68	0.64	0.45	0.63	0.65	0.76	0.55
	24	0.58	0.61	0.66	0.61	0.56	0.71	0.46
	48	0.57	0.56	0.60	0.55	0.54	0.44	0.44
	72	0.55	0.59	0.55	0.48	0.28	0.31	0.47
EDTA 4ºC	0	0.62	0.59	0.75	0.79	0.77	0.75	0.51
	24	0.62	0.68	0.73	0.71	0.74	0.69	0.48
	48	0.63	0.65	0.63	0.65	0.52	0.48	0.58
	72	0.61	0.63	0.56	0.51	0.60	0.40	0.68
