# Supplementary material for: Impact of sample processing delays on plasma markers of inflammation, chemotaxis, cell death, and blood coagulation
Source: PLoS One. 2024 Oct 31;19(10):e0311921. doi: 10.1371/journal.pone.0311921 (PMC11527306; doi:10.1371/journal.pone.0311921)
Supplement: S5 Table — (PDF) [file pone.0311921.s007.pdf]

Supplemental Table 5
Thrombin generation parameters in citrate plasma samples from ICU patients and healthy volunteers

	Time (h)	Volunteer 1	Volunteer 2	Volunteer 3	Volunteer 4	Volunteer 5	Volunteer 6
Lag Time RT (min)	0	4	3	4	3	4	5
	24	4	3	5	4	3	4
	48	3	4	5	4	3	5
	72	4	4	5	4	3	5
Lag Time 4ºC (min)	0	3	3	4	3	4	5
	24	3	3	4	4	3	4
	48	3	3	3	4	3	4
	72	4	4	3	4	3	4

	Time (h)	Patient 1	Patient 2	Patient 3	Patient 4	Patient 5	Patient 6	Patient 7	Patient 8
Lag Time RT (min)	0	4	7	4	7	17	6	3	3
	24	3	6	4	7	27	8	3	3
	48	3	8	4	8	29	9	3	3
	72	5	8	4	6	30	10	3	3
Lag Time 4ºC (min)	0	4	7	4	7	17	6	3	3
	24	3	6	3	7	18	6	3	3
	48	3	9	4	6	19	7	3	3
	72	4	7	3	7	18	7	3	3

	Time (h)	Volunteer 1	Volunteer 2	Volunteer 3	Volunteer 4	Volunteer 5	Volunteer 6
AUC RT (nM x min)	0	4248	4681	5380	4435	6230	4715
	24	3991	4367	4209	3968	5735	3831
	48	3925	4161	4022	3538	6497	3890
	72	4261	4355	4536	2699	5960	4143
AUC 4ºC (nM x min)	0	4248	4681	5380	4435	6230	4715
	24	5681	4329	5288	4951	5913	3831
	48	5850	4162	5266	5017	5997	3890
	72	5581	4445	5098	3692	6421	4143

	Time (h)	Patient 1	Patient 2	Patient 3	Patient 4	Patient 5	Patient 6	Patient 7	Patient 8
AUC RT (nM x min)	0	3931	1619	6487	2760	2880	2544	3753	3962
	24	3753	1359	6195	2049	1346	2202	4210	4361
	48	3688	1584	5187	1737	906	1667	4346	4194
	72	3063	1572	6202	1525	1270	1613	4375	4451
AUC 4ºC (nM x min)	0	3931	1619	6487	2760	2880	2544	3753	3962
	24	3259	1579	3723	1584	3331	2104	4849	4488
	48	3642	1593	5696	1895	3310	2429	4408	4350
	72	3438	1467	5488	2344	3025	1766	3994	4064
