# Supplementary material for: Impact of sample processing delays on plasma markers of inflammation, chemotaxis, cell death, and blood coagulation
Source: PLoS One. 2024 Oct 31;19(10):e0311921. doi: 10.1371/journal.pone.0311921 (PMC11527306; doi:10.1371/journal.pone.0311921)
Supplement: Attachment — Submitted filename: Response to reviewers_FINAL.docx [file pone.0311921.s008.docx]

April 24^th^, 2024

Dear PLOS One editorial team,

We would like to thank the reviewers for their detailed review of our manuscript entitled “**Impact of sample processing delays on plasma markers of inflammation, chemotaxis, cell death, and blood coagulation**”. We are grateful for their efforts to provide constructive feedback and we hope that our revised manuscript will address their concerns.

Our responses to the reviewers’ questions are below. We are submitting both a revised manuscript with tracked changes and a clean copy.

Sincerely,

Jennifer Tsang

**______________________________________________________________________**

***Reviewer #1:*** *Good study, well-written*

*Clarity required:*

*1. What determined the 72-hour cut-off?*

Response: A 72-hour cut-off was chosen to approximate the real-world situation of a blood sample being collected in a community hospital and shipped by courier to a central laboratory for processing and storage. Typical courier shipping takes 24-48 hours so 72 hours was chosen as the maximum processing delay to be one day beyond this period. We have added this information to the methods section.

*2. Why was IL-6 the only cytokine measured in citrate and EDTA tubes?*

Response: The multiplex assay allowed us to measure 4 cytokines and 6 chemokines simultaneously in each sample. For cost reasons, we were only able to perform the Multiplex assay using one anticoagulant. We chose EDTA as this is typically used in plasma protein studies. We also analysed IL-6 levels by ELISA in both EDTA and citrate tubes as IL-6 is the most frequently analysed cytokine in critical care studies and we wished to confirm that the results were independent of anticoagulant type.

*Suggested revision:*

*3. The Discussion needs to interrogate the literature more on IL-8 over time eg.
Patient 1 with a 10-fold increase in IL-8. Apart from vascular trauma, is there any other explanation, or other studies on IL-8 in ICU or vascular trauma?*

Response: We have expanded the third paragraph of the Discussion (p. 12) to provide additional details on plasma IL-8 levels.

IL-8 is a unique chemokine that is stored in the Weibel-Palade bodies (WPBs) of vascular endothelial cells and is released upon endothelial activation or injury [17,18]. Circulating IL-8 levels are increased in many ICU patients (including those with trauma, sepsis, burns, and acute pancreatitis), and they predict the development of multiple organ failure [19].

Vascular trauma can induce the release of bone marrow-derived endothelial precursor cells (EPCs) into the circulation [20]. Patient #1 was the only trauma patient in the cohort and was the only patient to show a change in IL-8 levels with delayed processing. One hypothesis is that circulating endothelial cells and/or EPCs resulting from the trauma promoted ongoing IL-8 production in the blood sample. Further studies will be required to confirm this hypothesis.

*4. The small sample size needs to be documented as a Study Limitation.*

Response: Thank you for making this important point. We have updated the discussion section of the manuscript to include the small sample size as a study limitation.

***Reviewer #2:*** *Impact of sample processing delays on plasma markers of inflammation, chemotaxis, cell death, and blood coagulation.*

*This manuscript describes a study of the effect of delayed processing times, temperature during delayed processing, and tube type on various biomarkers, including cytokines, chemokines, cell-free DNA, and thrombin (blood clotting potential). Samples were taken from a small number of healthy donors as well as ICU patients, and were either processed immediately or left for 24, 48, or 72 hours at either room temperature or 4^o^C. The authors report no effect of any of the treatment conditions on any of the biomarkers they measured and conclude that collecting samples and shipping them to a central location for processing should be acceptable for research studies.*

*Major concern:*

*1. Not all the assays were reported for all the treatments (other than thrombin, which the authors explained could not be done on EDTA tubes). Why is this? For example, Figure 1 is cytokines from ICU patients, EDTA tubes, room temperature and Figure 2 is chemokines from ICU patients, EDTA tubes, room temperature. For both of these figures, where are the data for healthy individuals, citrate tubes, and 4 degrees C? Without seeing all the data, it is impossible to confirm that the data support the conclusions.*

Response: Thank you for this feedback. We have added the results for all of the additional samples and conditions as supplementary tables. None of the tested samples or conditions resulted in significant changes in plasma protein or cfDNA values. This is noted in the text. In the main text we chose to display the results for ICU patients and EDTA tubes as this is the sample set most relevant to critical care studies.

*2. The study only included 7 healthy volunteers and 9 ICU patients. Is this enough subjects to be able to see an effect? A power calculation would be useful here.*

Response: Thank you for this question. Given the repeated measures design of the study, the sample size of 7 (healthy controls) or 9 (ICU patients) was sufficient to give > 80% power to detect a medium effect size of 0.5. It was not sufficient, however, to exclude a smaller effect size. We have added this observation to the methods section and to the study limitations.

*Other comments:*

*3. Where were the blood samples collected from the healthy volunteers? At the same location as for the patients?*

Response: The blood samples were collected from healthy volunteers at the Thrombosis and Atherosclerosis Research Institute (TaARI) research labs. The blood samples from the patients were collected from the medical-surgical and neurotrauma ICUs of the Hamilton General Hospital, which is connected to TaARI. This information has been added to the methods section.

*4. How many tubes total were collected from each participant? With 4 time periods and two temperatures, were there 8 EDTA and 24 citrate tubes collected? Or were there only 3 citrate and 1 EDTA tubes collected? If the latter, were the tubes aliquotted after they were collected and then put under the various time and temperature treatments? And if so, what was the aliquot protocol and what were they aliquotted into?*

Response: For each participant, the blood was collected into three citrate tubes (2.7mL each) and one EDTA tube (10mL). The blood in the pooled citrate tubes was aliquoted into 7 Eppendorf tubes (1mL each). Similarly, the blood in the EDTA tubes was aliquoted into 7 Eppendorf tubes (1mL each). The Eppendorf tubes were subsequently stored at either room temperature or 4°C for 0, 24, 48, 72 hours. The blood was then centrifuged (2500 x g for 15 min) and the plasma portion was stored in 60 uL aliquots at -80^o^C. We have updated the Methods section of the manuscript to provide these details.

*5. Were the exact type and number of tubes that were collected from the healthy subjects also collected from the ICU patients?*

Response: Yes, the type and number of tubes that were collected from the healthy subjects were identical to those collected from the ICU patients. We have clarified this in the methods.

*6. How were the two temperatures maintained? Were the room temperature samples left on a bench, or were they packed into a temperature controlled container? Were the 4C samples put in a refrigerator? Or packed in a cooler? If the latter, was there a temperature tracker included to confirm the samples stayed cold for the entire time? And was each set (by time-delay) packed separately?*

Response: The room temperature samples were left on the bench in the TaARI labs, which are temperature controlled at 23°C. The 4°C samples were stored in a temperature-controlled cold room (Honeywell Lab Works) at TaARI. The temperature in the cold room is tracked. We have updated the Methods section to provide these details.

*7. When were the assays run?/how long were the samples frozen prior to running the assays?*

Response: Samples were frozen at -80˚C immediately after processing. This is in keeping with how samples from critical care studies are handled. All assays were run within 3 months of sample collection. We have updated the Methods section to provide this information.

*8. Were the samples labeled in a masked fashion so that the laboratory staff did not know which sample had been treated which way?*

Response: Yes, the samples were labeled in a masked fashion. We have updated the Methods section to clarify this point.

*9. Were all samples per individual run together in the same assay batch?*

Response: Yes, all samples per individual were run together in batches. We have updated the Methods section to clarify this point.

*10. Were there any quality control samples included in the assays to ensure that the assays were running reliably?*

Response: Yes, quality control samples were included to ensure that the assays were running reliably. We have updated the Methods section to clarify this.

*11. Were any data collected from the healthy volunteers, such as age and sex?*

Response: Yes, data on age and sex were collected from the healthy volunteers. This information can be found on page 7 of the Results section. There were 4 male and 3 female volunteers with a mean age of 27 ± 7 years.

*12. Figure 3 is cfDNA for all treatments at room temperature, and Supplemental Figure 2 is all treatments at 4C. Why not show them together in one figure?*

Response: We have updated Figure 3 to include all of the treatments. Supplemental Figure 2 is now removed.

*13. There are some extreme values for some of the biomarkers. It would be nice for the authors to put these into context. What is considered the normal range and are the extreme values clinically relevant?*

Response: The new supplemental tables of cytokine and chemokine values include the normal ranges. We have updated the Discussion section to put some of the high values of biomarkers into context.

*14. Related to that, it is difficult to compare changes over time because some of the y-axis values are either shown with different scales, or so large that the changes cannot be seen. For example, Figure 2 IL-8, patient #1 is so extreme you can’t make out the variation for the other patients. And MCP-1, the scale is different for patient 3 compared with the other patients. Displaying the values of each biomarker at each time/temperature/tube in a table (perhaps supplemental) would be beneficial.*

Response: We have added four new Supplemental tables to show the biomarker data at each time/temperature/tube: Supplemental Table 2 (multiplex cytokines), Supplemental Table 3 (multiplex chemokines), Supplemental Table 4 (cell-free DNA), and Supplemental Table 5 (thrombin generation parameters).

***Additional Editor Comments:***

*Data Completeness and Presentation:*

*Reviewer 2 has raised concerns about the completeness of data presentation, specifically regarding the assays for all treatment conditions. Please ensure that all relevant data, including those for control conditions and different storage times and temperatures, are fully presented and discussed. This may involve adding supplementary material if necessary.*

Response: As requested by Reviewer 2, we have added four supplemental tables to show the biomarker data at each time/temperature/tube.

*Methodological Clarifications:*

*Clarify the rationale behind the 72-hour cut-off for sample processing delays. It is crucial to explain why this specific timeframe was chosen and how it aligns with the objectives of your study.*

Response: The objective of this study was to determine if delays in blood sample processing affect the stability of blood biomarkers. A 72-hour cut-off was chosen to approximate real-world situations where blood samples are collected in community hospitals and then shipped to a central site for processing and storage. Most courier services are able to ship samples within 1 or 2 days.

*Justify the decision to measure IL-6 as the only cytokine in both citrate and EDTA tubes. A detailed explanation will help strengthen the methodology section of your paper.*

Response: The multiplex assays allowed us to measure 4 cytokines and 6 chemokines in EDTA plasma samples from ICU patients at a cost of about $12,000 CAD. Due to limitations in our research budget, we decided to compare citrate versus EDTA plasma samples using a single immunoassay (the IL-6 ELISA kit from R&D systems) at a cost of about $900 CAD. We found that delayed sample processing did not affect IL-6 levels in healthy volunteers and ICU patients using either EDTA or citrate plasma samples. Similarly, we found that delayed sample processing (at room temperature or 4^o^C) did not affect cell-free DNA levels (Figure 3) or blood clotting parameters (Figure 4) in healthy volunteers and ICU patients using either EDTA or citrate plasma samples.

*Discussion and Literature Comparison:*

*The discussion section requires expansion to thoroughly interrogate the literature on IL-8 over time, particularly concerning patient 1 with a 10-fold increase in IL-8. Compare your findings with existing studies on IL-8 in ICU or vascular trauma contexts to provide a deeper analysis of your results.*

Response: As requested by Reviewer 1, we have updated the Discussion section of the manuscript to provide more references on L-8 levels.

*Address the small sample size by documenting it as a Study Limitation. Discuss how the sample size may affect the generalizability of your findings and any implications for future research.*

Response: We have updated the Discussion section with the following sentences:

“Another limitation of this study is the small sample size of the study participants. Future research could focus on a larger sample size as well as the inclusion of non-ICU patients which may help to increase the generalizability of our findings.”

*Data Availability Statement:*

*Address Reviewer 2's concern regarding the data availability statement. PLOS ONE requires authors to make all data underlying the findings described in their manuscript fully available without restriction. Please revise your Data Availability Statement to comply with this policy, or provide a more detailed explanation of any restrictions and how researchers can access the data if necessary.*

Response: As requested by Reviewer 2, we have added four new supplemental tables to show the biomarker data at each time/temperature/tube: Supplemental Table 2 (multiplex cytokines), Supplemental Table 3 (multiplex chemokines), Supplemental Table 4 (cell-free DNA), and Supplemental Table 5 (thrombin generation parameters).

*Additional Clarifications and Information:*

*Provide more details on how blood samples were collected and processed, including information on maintaining two temperatures, the type and number of tubes collected from each participant, and specifics on the aliquot protocol.*

Response: As requested by Reviewer 2, we have updated the Methods section to provide more details on the blood processing protocol, including the types and number of tubes collected from each participant and how the plasma was aliquoted prior to freezing.

*Include information on the timing of assays and whether samples were labeled in a manner that prevented the laboratory staff from knowing the treatment conditions, to ensure the integrity of the data.*

Response: We have updated the Methods section to explain the timing of assays. We have also clarified that samples were masked and processed in batches to ensure blinding of laboratory staff.
