# Supplementary material for: Impact of sample processing delays on plasma markers of inflammation, chemotaxis, cell death, and blood coagulation
Source: PLoS One. 2024 Oct 31;19(10):e0311921. doi: 10.1371/journal.pone.0311921 (PMC11527306; doi:10.1371/journal.pone.0311921)
Supplement: Attachment — Submitted filename: Response to Reviewers - Second revision.docx [file pone.0311921.s009.docx]

August 19^th^, 2024

Dear PLOS ONE editorial team,

We thank the reviewers and the editorial team for reviewing our revised manuscript entitled **“Impact of sample processing delays on plasma markers of inflammation, chemotaxis, cell death, and blood coagulation”**. Our responses to the reviewers’ questions are provided below. We are submitting a revised copy of the manuscript with tracked changes as well as a clean copy.

We thank the reviewers for their helpful feedback and comments. We hope that we have addressed their concerns appropriately.

***Reviewer #1:*** *All comments have been addressed.  Corrections accepted, for choice of 72 hour time frame, IL-6 measurement in citrate and EDTA tubes and the sample size. The Discussion around the IL-8 increase improves the objectivity of the paper.*

***Reviewer #2:***  *The authors have done a lot of work to address my comments. Some lingering items:*

*1. The authors have added additional results in supplemental tables. However, I still cannot find the following:*

*a. Cytokines, EDTA, ICU patients, cold storage*

*b. Cytokines, EDTA, healthy participants, room temp & cold storage*

*c. Cytokines, citrate, ICU patients room temp & cold storage*

*d. Cytokines, citrate, healthy participants, room temp & cold storage*

*e. Chemokines, EDTA, ICU patients, cold storage*

*f. Chemokines, EDTA, healthy participants, room temp & cold storage*

*g. Chemokines, citrate, ICU patients room temp & cold storage*

*h. Chemokines, citrate, healthy participants, room temp & cold storage*

*i. Also: IL-6, citrate, healthy participants is missing from Supplemental Figure 1*

*Were these assays run but not reported? Or not run? The methods section should specify exactly what assays were run on what conditions, and if the above assays were not run, then indicate why.*

Response: Thank you for this question. Due to budget limitations, we could only perform the multiplex assay in one set of samples. We chose the ICU patient samples because we felt they were more clinically-relevant and the EDTA samples because EDTA is typically used as the anticoagulant in proteomics studies. We also chose the room temperature samples, rather than 4˚C samples, because we felt they were more likely to show a change with delayed processing and reflected the scenario in which samples are mailed without ice. We have edited the Methods section to clarify these issues.

*2. Figures 1, 2, and 4 seem to be missing so I was not able to compare the text and the figures (although if the data have not changed from the original version then I do not need to re-review these).*

Response: We apologize for any confusion. The figures were included with the original submission but were left out of the resubmission as they were unchanged. We are including them again with this submission.

*3. How many quality control samples were included in the batches, and can you report the coefficient of variation (CV) or another statistic to measure the reliability of the assays? Also, were quality control samples included in all assays, or only the IL-6 and cfDNA assays?*

Response:  Thank you for this question. For the cfDNA and IL-6 assays, we included both “high” and “low” quality control samples.  The intra- and inter-assay CV for the cfDNA assay were 3.7% and 14%, respectively.  The intra- and inter-assay CV for the IL-6 ELISA were 2.6% and 4.5%, respectively.

For the multiplex cytokine and chemokine assays, two normal plasma samples were included as quality control samples.  The average intra- and inter-assay CV for the multiplex cytokine assay were 3.5% and 7.6%, respectively. The average intra- and inter-assay CV for the multiplex chemokine assay were 7.9% and 5.1%, respectively.

For the thrombin generation assay, a normal pooled plasma sample was included in each run.  The intra-assay CV for the lag time and area under the curve (AUC) were 7.9% and 5.2%, respectively.  The inter-assay CV for the lag time and area under the curve (AUC) were 10.2% and 12.1 %, respectively.

We have updated the manuscript to include the CV values for each assay.

*4. The authors indicate that they had greater than 80% power to detect a “medium effect size of 0.5”. What are the units of this 0.5? and what does “medium” mean?*

Response: Thank you for this question. Our original sample size calculation was based on a paired t-test design comparing an individual cytokine or chemokine in paired samples after 0 vs 72 hours of sample processing delay. Based on this calculation, a sample size of ≥ 7 patients is sufficient to give 80% power to detect a “medium” effect size (Cohen’s d) of 0.5 with an α error rate of 0.05. Cohen’s d is a unitless measure calculated from the anticipated difference between the group means divided by the standard deviation. An effect size of 0.2 is considered “small”, 0.5 is “medium”, and 0.8 is “large”. In the revised version of the manuscript, we have included the sample size calculation for a repeated measures ANOVA design. Using this experimental design, ≥6 samples is sufficient to give a Cohen’s f (effect size) of 0.17, which is considered a small to medium effect size for a repeated measures experiment. Sample size calculations were conducted using the G*power software.
